# Detection of Patients with Congenital and Often Concealed Long-QT Syndrome by Novel Deep Learning Models

**DOI:** 10.3390/jpm12071135

**Published:** 2022-07-13

**Authors:** Florian Doldi, Lucas Plagwitz, Lea Philine Hoffmann, Benjamin Rath, Gerrit Frommeyer, Florian Reinke, Patrick Leitz, Antonius Büscher, Fatih Güner, Tobias Brix, Felix Konrad Wegner, Kevin Willy, Yvonne Hanel, Sven Dittmann, Wilhelm Haverkamp, Eric Schulze-Bahr, Julian Varghese, Lars Eckardt

**Affiliations:** 1 Department for Cardiology II-Electrophysiology, University Hospital Münster, 48149 Münster, Germany; lea-philine.hoffmann@t-online.de (L.P.H.); benjamin.rath@ukmuenster.de (B.R.); gerrit.frommeyer@ukmuenster.de (G.F.); florian.reinke@ukmuenster.de (F.R.); patrick.leitz@ukmuenster.de (P.L.); antonius.buescher@ukmuenster.de (A.B.); fatih.guener@ukmuenster.de (F.G.); felix.wegner@ukmuenster.de (F.K.W.); kevin.willy@ukmuenster.de (K.W.); lars.eckardt@ukmuenster.de (L.E.); 2 Institute of Medical Informatics, University of Münster, 48149 Münster, Germany; l_plag03@wwu.de (L.P.); tobias.brix@ukmuenster.de (T.B.); julian.varghese@ukmuenster.de (J.V.); 3 Institute for Genetics of Heart Diseases (IfGH), University Hospital Münster, 48149 Münster, Germany; yvonne.hanel@ukmuenster.de (Y.H.); sven.dittmann@ukmuenster.de (S.D.); eric.schulze-bahr@ukmuenster.de (E.S.-B.); 4 Department of Internal Medicine and Cardiology, Charité University Medicine, 10117 Berlin, Germany; wilhelm.haverkamp@charite.de

**Keywords:** electrophysiology, long-QT syndrome, ECG, artificial intelligence, deep learning models

## Abstract

Introduction: The long-QT syndrome (LQTS) is the most common ion channelopathy, typically presenting with a prolonged QT interval and clinical symptoms such as syncope or sudden cardiac death. Patients may present with a concealed phenotype making the diagnosis challenging. Correctly diagnosing at-risk patients is pivotal to starting early preventive treatment. Objective: Identification of congenital and often concealed LQTS by utilizing novel deep learning network architectures, which are specifically designed for multichannel time series and therefore particularly suitable for ECG data. Design and Results: A retrospective artificial intelligence (AI)-based analysis was performed using a 12-lead ECG of genetically confirmed LQTS (*n* = 124), including 41 patients with a concealed LQTS (33%), and validated against a control cohort (*n* = 161 of patients) without known LQTS or without QT-prolonging drug treatment but any other cardiovascular disease. The performance of a fully convolutional network (FCN) used in prior studies was compared with a different, novel convolutional neural network model (XceptionTime). We found that the XceptionTime model was able to achieve a higher balanced accuracy score (91.8%) than the associated FCN metric (83.6%), indicating improved prediction possibilities of novel AI architectures. The predictive accuracy prevailed independently of age and QT_c_ parameters. Conclusions: In this study, the XceptionTime model outperformed the FCN model for LQTS patients with even better results than in prior studies. Even when a patient cohort with cardiovascular comorbidities is used. AI-based ECG analysis is a promising step for correct LQTS patient identification, especially if common diagnostic measures might be misleading.

## 1. Introduction

The congenital long-QT syndrome (LQTS) is the most common cardiac channelopathy with an incidence of 1:2000–3000 [1,2]. LQTS is commonly associated with a combination of QT_c_-interval prolongation and clinical manifestations such as syncope or sudden cardiac death [1,2]. The ECG phenotype of the disease results from mutations in various cardiac ion channel genes with clinical and electrocardiographic (ECG) manifestations depending on the degree of functional ion channel disturbance and subsequent reduction in the cardiac repolarization reserve [1,2,3]. Until now, the diagnosis of LQTS relies on genetic testing and the Schwartz Score, taking into account ECG and clinical parameters [1]. Specific ECG parameters (e.g., T-wave morphologies [4], QT-interval changes upon stress (e.g., epinephrine or treadmill QT-stress test [5,6]) or echocardiographic markers can help to unmask LQTS but still lack relevant diagnostic value [7,8,9]. Hence, reasonable simple tests are still lacking, leaving the resting 12-lead ECG as the most essential diagnostic tool for LQTS detection [10,11,12].

Furthermore, LQTS mutation carriers may present with a normal baseline ECG and repolarization (so-called concealed LQTS or non-penetrant LQTS) [13], making the identification and diagnosis of LQTS difficult for physicians in daily practice [10,11,12] with over 40% of patients with LQTS having a normal QT_c_ at rest [14]. Hence, there is a high risk of underdiagnosing LQTS. This indicates the necessity for improving LQTS diagnosis. We therefore intended (1) to validate prior attempts for identification of concealed LQT with our more generalizable patient cohort with additional major comorbidities present, also using statistical matching filtering out deceptive cohort differences, and (2) to test performance advantage using a modern network architecture, which has been optimized especially for ECG-like data.

## 2. Methods

### 2.1. Study Cohort

In this single-center study, consecutive patients presenting at our clinic with a genetically proven (*n* = 124) congenital LQTS (LQTS 1: 65 patients; LQTS 2: 44 patients; LQTS 3: 12 patients; LQTS 5: 3 patients) including 41 concealed cases (33%) and a control cohort extracted from an existing patient registry (*n* = 161) without known congenital LQTS or a history of QTc-prolonging drugs, but any other possible cardiac or non-cardiac disease were included to develop and validate data sets used in this AI-based analysis of ECG raw data. Concealed LQTS cases were defined by the presence of an LQTS mutation, but a normal repolarization based on the resting ECGs, i.e., with a QTc interval <460 ms for women (*n* = 20) or <450 ms for men (*n* = 21).

The control cohort was extracted randomly from our electronic medical record system. These patients included in- and outpatients of any department for internal medicine and were consequently analyzed for the aforementioned inclusion and exclusion criteria.

All 12-lead ECGs were performed with the patient lying in a supine position with a sampling rate of 500 Hz using GE systems, consequently saving its raw data (xml format) in its own MUSE data management system (GE Healthcare, Chicago, IL, USA) for later retrieval. ECG data of patients with genetically proven LQTS and a control cohort were extracted from the electronic medical record system and the MUSE data management system. This resulted in a total data set of 124 patients (165 ECGs) with genetically proven LQTS and 161 (565 ECGs) control patients with no known channelopathy and no history or current intake of QT-prolonging drugs at the time of ECG retrieval. QTc is calculated by the Bazett Formula using RR-Interval and the QT-Interval. This study was reviewed and approved by the local ethics committee.

### 2.2. Outcomes

The primary outcome of our study was to confirm the potential of analyzing raw ECGs to predict congenital LQTS. Included were patients with congenital LQTS as well as a control cohort having a wide spectrum of patients with no history or suspicion of LQTS or QT-prolonging drug intake. Yet, any other cardiac or non-cardiac disease was possible. Both were analyzed by AI-based methods.

Hence, we investigated the capabilities of modern network architectures to increase predictive accuracy compared to traditional deep learning approaches. We examined these using various performance metrics such as sensitivity, specificity, balanced accuracy score, *F*_1_ score, and area under the curve (AUC).

### 2.3. Statistical Analysis

To show that the AI system does not only perform well based on differences in simple QTc and age values, we also implemented a strict subset of our data matched by QTc and age. To determine this, we utilized a random under-sampling approach—each ECG from the LQTS group is randomly assigned to an ECG from the control group, provided that this measurement was within a tight interval in terms of age (±2 years) and QTc (±10 ms). If no suitable ECG was found or if it was already assigned to another, it could not be included in the matching process. In this way, the performance of our AI-based classification could be evaluated independently of the parameters age and QTc. To learn from as many samples as possible, we trained each convolutional neural network (CNN) model on all training samples. As a consequence of the subsampling, we considered all performance metrics for two different sets, one for the complete test set; the other for the intersection between the test and matched subset.

Statistical differences between the groups (with respect to both sets) were calculated using the Mann–Whitney U-rank test for continuous data, χ^2^ test for categorical data, or Barnard’s exact test if the total number of samples was too small. To ensure independence, all tests were calculated on a per-patient basis; for this purpose, the averaged ECG-based values were considered.

### 2.4. Machine Learning

It has already been shown that CNNs provide high accuracy in pattern recognition on ECG time series [15]. In addition to the behavior of a classical fully convolutional network (FCN), we examined the performance of a more advanced architecture, which is particularly well-suited for multichannel time series data: The XceptionTime model published in 2019 [16] was developed for the classification of multichannel surface electromyography (sEMG) signals. In this work, four XceptionTime modules are connected in series to capture both the temporal and spatial information of the multivariate signal. The problem is transferable to a 12-lead ECG measurement. In FCN models, leads are investigated individually via predefined one-dimensional kernels, and only afterwards, the extracted information of all leads is merged. In contrast, the XceptionTime model is based on a simultaneous analysis of multiple leads and different sized kernels to address both long- and short-time intervals.

To prevent identity confounding [17], we used a grouped version of 5-fold cross-validation (randomized over 5 replicates): ECGs of the same patient were grouped such that samples of one patient will not occur both in the training and test set. In this way, we ensured that we assessed the quality of the model based only on individuals who had not been seen in the training process. In addition to this, we split up a validation set of 10% in order to implement an early stopping criterion. This results in a distribution of 70% train, 10% validation, and 20% test set. In this method, the matched subset was randomly assigned to the splits. For each sample, the unfiltered raw data were used as input, resulting in a 12 × 5000 matrix with a time series for each of the 12 leads. Each model was trained with a balanced cross-entropy loss over a maximum number of 150 epochs and is based on the Python package PyTorch.

In addition to the described CNN analyses on the raw ECG data, we determined the performance of a simplified machine learning classification based on the parameters QTc, age, and biological sex. This illustrated the diagnostic possibility based on these parameters on our data set. For this purpose, we used a balanced support vector classifier (SVC) of the Python package scikit-learn.

## 3. Results

Baseline characteristics of our LQTS and control cohorts are presented as statistical analysis using R-statistics in Table 1. Our LQTS cohort included 41 patients (33%) with a concealed phenotype of the disease (male: *n* = 21 (17%); female: *n* = 20 (16%)). Median Schwartz Score for the overall LQTS cohort was 4 (±1.8), for the manifest LQTS cohort 5 (±1) and for the concealed cohort 3 (±1.8). Our healthy cohort had a median Schwartz Score of 0.84 (±1.1).

**Figure 1:**
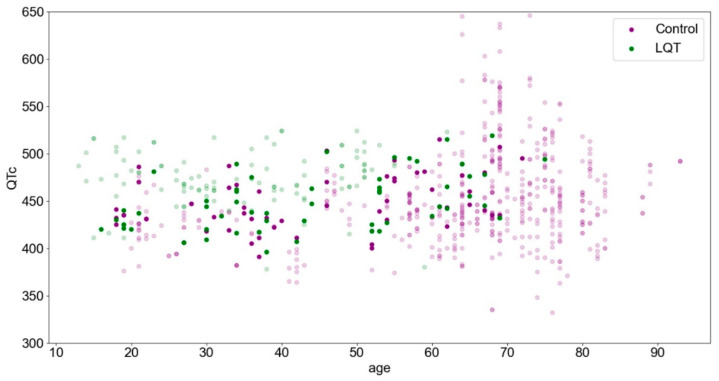
The study cohort is presented according to age and QTc parameters. The highlighted points represent the matched subset.

The summary of the studied cohorts showed an imbalance with respect to the mean age (60 ± 18 vs. 38 ± 15 years). While the mean QT_c_ values of both groups were close with 453 ± 50 ms and 465 ± 32 ms, we observed a smaller QT_c_ value, especially in the younger control subjects; therefore, in addition to age, we also considered the QT_c_ for the matching process. Thus, statistically, we subsampled the LQTS and control groups from the same distribution with respect to age and QT_c_ (*p* ≥ 0.71). This process is illustrated in Figure 1.

Although the overall cohort is more characterized by LQTS at lower ages in association with a higher QT_c_ (upper left), control patients are more likely to be found at higher ages (right). This trend is no longer evident in the matched subset.

The overall results of our analysis are listed in Table 2.

The XceptionTime model outperformed the FCN and the simplified SVC approach with a balanced accuracy score of 91.8%, compared to the associated values of 83.6% (FCN) and 79.2% (SVC). Although the simplified machine learning classification via SVC achieved this performance based on the three parameters age, QT_c_, and sex, it decreased down to barely 53.2% for the matched test subset. This trend was hardly observed in the CNN approaches, where both performances were almost equal (the difference was less than 1.5% according to the balanced accuracy score); however, the standard deviation increased for the subset for each model, which was attributable to the much smaller sample size.

The AUC averaged over 25 cross-validation splits was at or above 0.9 for both CNN models (FCN: 0.9, XceptionTime: 0.97). Furthermore, only for the XceptionTime model, we observed a balanced ratio between specificity and sensitivity in both test sets, with all values reaching at least 90%. Similar results are observed with the *F*_1_ score, such that the more complex XceptionTime model is 17.2% more accurate than the FCN on the complete test set.

## 4. Discussion

QT_c_ prolongation represents an independent risk factor for sudden cardiac death and is considered a predictor of all-cause cardiovascular mortality. Its correct measurement is very important in everyday clinical practice [18]. Many studies have shown that patients having a QT_c_ of ≥500 ms at the time of admission have a 2 to 4-fold increased risk of death; hence, it seems an important predictor of all-cause mortality, potentially outperforming many comorbidity indices [18,19]. Moreover, optimally treated, only a small number of patients with LQTS will develop relevant cardiac arrhythmias making the early diagnosis and treatment of this cohort crucial [20]; however, given that a substantial number of these patients do not present with QT_c_-prolongation as its most pathognomic feature, the need to optimize current clinical practices (e.g., QT interval measurement), as well as the development of further diagnostic tools, seems essential. We herein present CNN models able to distinguish ECG of patients with a genetically proven LQTS, including a large proportion of patients with a concealed LQTS, from that of a control cohort with no known LQTS or history of QT-prolonging medication but any other comorbidity possible. In addition, we highlight the importance of modern CNN models reaching even higher sensitivity and specificity in this regard, and lastly, possibly leading to an improved diagnosis and reducing the risk of over and underdiagnosing.

As it has already been established by Bos et al. [21] in their CNN analysis of ECG raw data of genetically proven LQTS patients, AI was able to distinguish between patients with congenitally proven LQTS from control patients with a sole suspicion for the disease with an AUC of 0.863 (95% CI, 0.824–0.903); however, as this model has only been validated against a highly selected patient cohort, the generalizability of this model has not been demonstrated, yet [21]. An AI model designed to detect ECG patterns associated with QTc prolonging medication was designed by Prifti et al. [22], demonstrating correct identification of patients taking sotalol and with a congenital LQT2 subtype with an ROC-AUC curve of 0.98 [23]. This cohort was well suited for the selected scientific question of detecting potassium channel block either by sotalol or by a congenital channel mutation (LQT2); however, with patients having an indication for antiarrhythmic treatment, a compromised generalizability of this control cohort cannot be assumed and should be further validated.

Moreover, as specific T-wave morphologies have been described and verified as viable diagnostic markers for the detection of congenital LQTS [4,8,24], Hermans et al. [25] developed a machine learning support vector model showing a high capacity for LQTS detection from genotype negative family members (AUC up to 0.901) by only viewing T-wave morphology without including the complete ECG-waveform.

In the present study, we were able to train a deep learning algorithm comparable to algorithms from Bos et al. [21] as well as Prifti et al. [22]. Furthermore, we aimed to validate these results with a more generalizable and comorbidly diseased control cohort as well as a modern CNN model (XceptionTime), especially well-suited for ECG-like data. As a consequence of the different nature of the diseased control cohort, because of its overall older age with accompanying typical cardiovascular diseases, we performed an additional subgroup analysis in which indirect learning based on age was no longer possible. Although age is not a direct component of the input parameters of the deep learning models, the ECGs show age-related differences to some extent [26]. Further, and also of note, by additionally matching the QT_c_ parameter, we were able to show a similarly good predictive performance (balanced accuracy score: 91.2%) compared to an unmatched model (balanced accuracy score: 91.8%). Hence, avoiding possible misclassification based on QT_c_-interval.

The field of those neural networks is a very fast-moving one—due to the increasing computational capacity—leading to ever new network architectures that may improve their ability to solve problems through specific calculation layers. For this reason, we took a closer look at a particular one of these architectures and compared it with the general FCN. We chose the XceptionTime model since it is possibly more suited for temporal and spatial analysis of multi-lead time series data.

We aimed at highlighting the possibilities of new architectures—and consequently the usage of AI in general—in the field of LQTS diagnostics and possibly aid in optimal and early diagnosis with subsequent treatment of these patients.

Using FCN models used in major prior studies [21,22], we were able to recreate results proving our AI model is as (or even more) able in the detection of patients with LQTS, including a large proportion of patients with a concealed phenotype, with an AUC of 0.9 showing stability over 25 cross-validation splits. It has to be highlighted that we reached these results despite the fact that our control cohort was even more generalized, including patients with major cardiac and other comorbidities; thus, we can further validate and highlight the potency of this diagnostic tool and the possibility of its implementation in everyday clinical practice. Furthermore, using modern CNN models more suited for time series raw data (XceptionTime model), we were able to achieve an even more improved balanced accuracy score of 91.8% on our data set, compared to the associated metric of the FCN with 83.6%. Hence, we sought to highlight the importance of the adequate and specific selection of AI models depending on the types of raw data used and the diagnostic purpose it is aimed to aid; thus, helping physicians in the correct diagnosis and treatment of these patients and serving as a possible true screening tool in the future. We are optimistic that specific hyperparameter adaptation, new architectures, or even larger patient collectives will push the boundaries here once more. Besides these optimizations, further points are pending to bring the algorithms closer to clinical application. In addition to the calibration of prediction probabilities for risk stratification or analyses over larger multicenter collectives, prospective studies investigating the benefit of AI for LQTS detection are particularly worth mentioning here.

## 5. Limitations

AI models supply a useful and economical diagnostic tool in comparison to more expensive or time-consuming diagnostic measures (e.g., genetic testing, echocardiography, MRI). This model evaluation does not represent a real-time clinical setting and thus requires further prospective clinical analysis. This work presents a single-center validation; further external validations by other heart rhythm clinics with patients of similar disease should be conducted in the future. The selection of our control cohort was based on a missing suspicion for LQTS or other cardiac channelopathy and thus could limit the generalizability of this model as a result of selection bias by the investigator. For this, a blinded patient inclusion in the setting of a prospective clinical trial would be best suited. Further, despite the overall low incidence of the disease, the presence of some concealed LQTS in the control cohort may be possible as they did not undergo genetic testing.

## 6. Conclusions

As LQTS can often be concealed, making the diagnosis and treatment for physicians difficult, we sought to validate priorly used as well as novel deep learning algorithms. To increase generalizability, we used a control cohort of patients with no suspicion for LQTS and no history of QTc-prolonging medications. Firstly, we can confirm that the FCN model achieved similar results in this regard despite our control cohort having numerous comorbidities. Secondly, the more advanced XceptionTime model reached even higher sensitivity and specificity, underlining the importance of adequate CNN model selection depending on the used data and scientific question. Of course, clinical settings cannot be compared to an AI model; however, as this goes against current practice and literature, further evaluation in prospective clinical trials should be performed to validate these results in a clinical setting.

## Figures and Tables

**Table 1:** Baseline characteristics of the study cohort separated into the LQTS, control group, and the corresponding subgroups matched according to age and QTc. LVEF, left ventricular ejection fraction; ICM, ischemic cardiomyopathy; NICM, non-ischemic cardiomyopathy; ATH, arterial hypertension; DM, diabetes mellitus; LQTS, long-QT syndrome.

	Control Group	LQTS Group	Matched Control Group	Matched LQTS Group	*p* Value, Control vs. LQTS Group	*p* Value, Matched Control vs. Matched LQTS Group
Number of Individuals	161	124	47	50		
ECGs, *n*	565	165	58	58		
Age (y), mean ± SD	60 (±18)	38 (±15)	44 (±16)	44 (±16)	<0.01	0.71
Male, *n* (%)	330 (58)	48 (29)	27 (47)	23 (40)	<0.01	0.77
Weight (kg) mean ± SD	84 (±16)	72 (±17)	82 (±21)	71 (±17)	<0.01	0.05
Height (cm) mean ± SD	178 (±10)	171 (±10)	174 (±12)	170 (±12)	<0.01	0.14
QTc (ms) mean ± SD	453 (±50)	465 (±32)	448 (±29)	449 (±30)	<0.01	0.97
LVEF (%) mean ± SD	56 (±11)	69 (±9)	54 (±13)	64 (±9)	<0.01	<0.01
ICM, *n* (%)	60 (37)	1 (1)	8 (17)	0 (0)	<0.01	0.04
NICM, *n* (%)	45 (28)	2 (2)	4 (9)	0 (0)	<0.01	0.06
ATH, *n* (%)	91 (56)	3 (2)	18 (38)	3 (6)	<0.01	<0.01
DM, *n* (%)	67 (42)	1 (1)	6 (13)	1 (2)	<0.01	0.07

**Table 2:** Performance metrics of the three considered classifiers in discriminating an ECG measurement from a patient with congenital LQTS from the control cohort depending on the complete or matched test set (mean (±SD) of 25 cross-validation splits). Cohorts were matched according to QTc and Age. AUC, area under the curve; SVC, support vector machine; FCN, fully convolutional network.

Classifier (Parameters)	Test Set	AUC	Balanced Accuracy	Specificity	Sensitivity	*F*_1_ Score
SVC (age, QTc, biological sex)	complete	0.9 (±0.03)	79.2% (±3.6)	74.6% (±8.2)	83.9% (±6.6)	62.5% (±6.7)
	matched	0.56 (±0.12)	53.2% (±10.4)	35.8% (±12.6)	70.6% (±15.5)	59.2% (±11.9)
FCN (raw ECG data)	complete	0.9 (±0.03)	83.6% (±4.1)	82.6% (±7.5)	84.7% (±8.2)	66% (±12)
	matched	0.88 (±0.08)	82.5% (±6.4)	78.4% (±13.2)	86.6% (±11.6)	82.6% (±10.6)
XceptionTime (raw ECG data)	complete	0.97 (±0.02)	91.8% (±2.8)	92.9% (±3.9)	90.8% (±5.7)	83.2% (±6.5)
	matched	0.97 (±0.04)	91.2% (±6.0)	92.5% (±9.9)	90.0% (±8.8)	89.4% (±11.1)

## Data Availability

The data presented in this study are available on request from the corresponding author. The data are not publicly available due to privacy.
